# Identification of differentially expressed miRNAs in the fatty liver of Landes goose (*Anser anser*)

**DOI:** 10.1038/s41598-017-16632-7

**Published:** 2017-11-24

**Authors:** Fang Chen, Hao Zhang, Jinjun Li, Yong Tian, Jing Xu, Li Chen, Jintao Wei, Na Zhao, Xuehai Yang, Wei Zhang, Lizhi Lu

**Affiliations:** 1 Institute of Animal Husbandry and Veterinary Science, Hubei Academy of Agricultural Sciences/Hubei Key Laboratory of Animal Embryo Engineering and Molecular Breeding, Wuhan, 430064 PR China; 2 Institute of Animal Husbandry and Veterinary Science, Zhejiang Academy of Agricultural Sciences, Hangzhou, 310021 PR China

## Abstract

Unlike mammals, in palmipedes de novo lipogenesis from diet takes place mostly in the liver. The French Landes Goose is famous for its high capacity and susceptibility to fatty liver production. While miRNAs play a critical role in the posttranscriptional regulation of gene expression, miRNAs that are involved in the regulation of goose hepatic steatosis have yet to be elucidated. Using high-throughput sequencing, we analyzed miRNAs expression profile of Landes goose liver after overfeeding for 21 days. Aan-miR-122-5p was the most frequently sequenced known miRNA, but it was unchanged after overfeeding. Compared with normal liver, we identified that 16 conserved miRNAs were up-regulated while the other 9 conserved miRNAs were down-regulated in fatty livers. Many of their predicted target genes played key roles in metabolic pathways leading to the development of hepatic steatosis in the goose by KEGG pathways analysis. *ACSL1* and *ELOVL6* were critical genes in hepatic lipid metabolism and had opposite expression patterns with aan-miR-203a and aan-miR-125b-5p, respectively. And we validated that aan-miR-203a and aan-miR-125b-5p might involve in the regulation of hepatic lipid metabolism by targeting *ACSL1* and *ELOVL6*, respectively. These results add to our current understanding of the regulation network in goose lipid metabolism.

## Introduction

The livers of animals are important tissues of lipid metabolism. In humans, hepatic steatosis is typically the causal factor for abnormal liver function and is frequently correlated with a variety of metabolic disorders, such as obesity, insulin resistance, and type II diabetes^1^. In contrast, in wild palmipedes under natural conditions, hepatic steatosis occurs as a consequence of evolutionary adaption to accumulated reserves needed for migration^2^. In poultry production, this specific capacity is exploited for the production of “foiegras”^3^. Geese form fatty livers after feeding on a carbohydrate-rich diet for less than two weeks. And the liver weight may increase more than 10-fold and up to 10% of BW^4^. The French Landes Goose is famous for its high capacity and susceptibility to fatty liver production.

In order to find the mechanism of the development of fatty liver, some researchers have studied the crucial genes involved in hepatic steatosis of waterfowl^2,5^. MicroRNAs (miRNAs) are 20–24-nt long, endogenous small noncoding RNAs. They can hybridize to sites in the mRNAs of protein-coding genes to direct posttranscriptional regulation of gene expression by inhibiting translation and/or inducing degradation of target mRNAs^6,7^. To date, evidences indicate that miRNAs are key trans-acting factors and a single miRNA can repress the production of hundreds of protein coding target genes through relatively mild means^7^. Dysregulated expression of miRNAs has been reported in human fatty liver production^8,9^. And many miRNAs have been well characterized for their roles in regulating lipid metabolism, such as miR-122^10^. Nevertheless, so far there is a scarcity of information available regarding the abnormal expression miRNAs and their target genes in fatty liver of goose. Thus, the studies on lipid metabolism-related miRNAs and their mechanisms in goose liver could serve as an important reference for poultry breeding and human liver disease research.

The goal of this study was to identify differentially expressed miRNAs in the fatty liver of the Landes goose by using high-throughput technologies, deep-sequencing analysis, and qRT-PCR. Furthermore, we have predicted the target genes of the miRNAs and preliminarily validated some miRNA-mRNA pairs involved in the development of goose fatty liver. These findings provided insights into the role of miRNAs in the hepatic steatosis of goose.

## Results

### Effects of overfeeding

**Figure 1:**
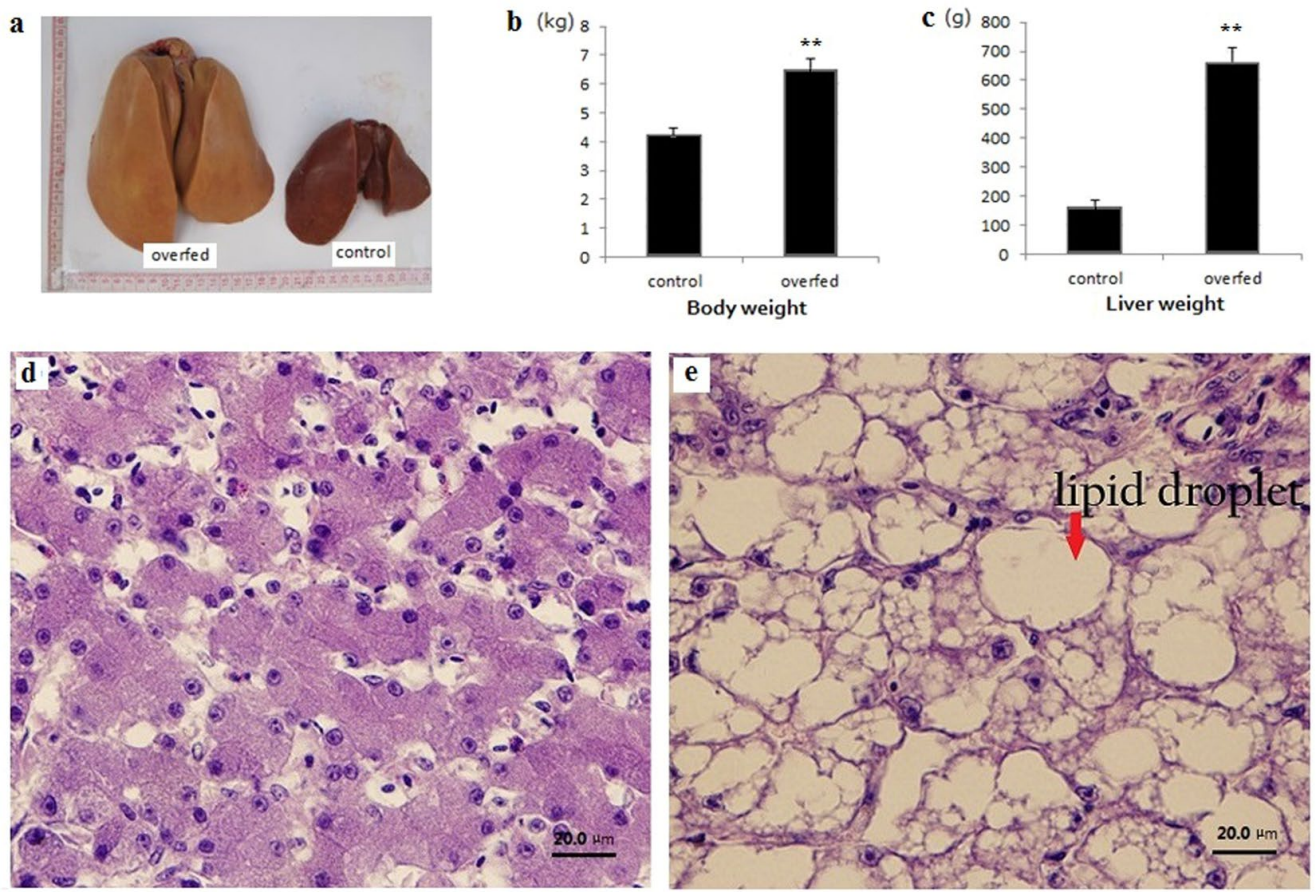
Effects of overfeeding. (**a**) Comparison of livers; (**b**) Comparison of body weight; (**c**) Comparison of liver mass; (**d**) Histology observation of normal goose liver (400×); (**e**) Histology observation of goose liver after overfeeding (400×). **Means *P* < 0.01.

As shown in Fig. 1, the body weight and liver weight of overfed geese increased 1.5-fold and 4.0-fold (*P* < 0.01), respectively. The liver accounted for 3.92% of total body weight in the control geese and 10.17% in overfed geese. The increased liver weight accounted for 21.80% of total increase in body weight. By histological examination, the livers from control geese and overfed geese displayed marked differences. In Fig. 1d, the cell boundaries of hepatocytes were visible, while the nuclei of hepatocytes were centrally located. However, the cell boundaries were blurry, as shown in Fig. 1e. In contrast, we clearly observed many lipid droplets, and the nuclei were compressed to the edges of the cells.

### miRNA expression profiles in *A. anser* liver

**Figure 2:**
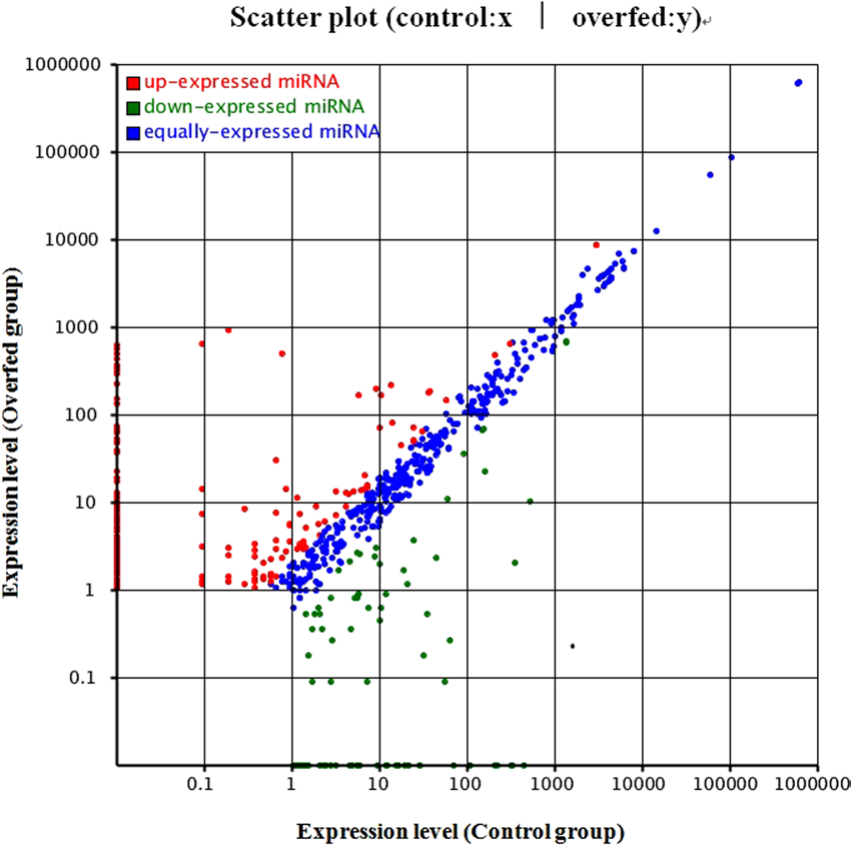
Scatter plot of miRNAs. Each point in the figure represents a miRNA. Red points represent up-regulated miRNAs (fold-change > 2, *P* < 0.001), green points represent down-regulated miRNAs (fold-change < 0.5, *P* < 0.001), and blue points represent no significant changed miRNAs (0.5 < fold-change < 2).

By sequencing analysis, the distributions of all small RNA classes in the two groups were displayed in Table S1. The information of a total 1410 miRNAs (995 from control group library, 1021 from overfed group library and 606 in both two groups) were obtained and their scatter plot were analyzed (Fig. 2). We listed some miRNAs, which showed expression with high predominance in Table 1. Of these miRNAs, the most frequently sequenced known miRNA was aan-miR-122-5p, which represented 70.34% and 72.01% of total miRNA reads in the control and overfed groups, respectively. However, the expression of aan-miR-122-5p was unchanged after overfeeding. In order to elucidate the role of miRNAs in the hepatic steatosis of geese, we compared miRNAs expression between normal livers and fatty livers. As shown in Table 2, 25 known conserved miRNAs were found differentially expressed, among which 16 conserved miRNAs were up-regulated while the other 9 conserved miRNAs were down-regulated in fatty liver.

**Table 1:** Expression predominance of miRNAs in the two groups. The list of miRNAs is arranged in the order of the expression in control group. The order of top 10 miRNAs in overfed group is a little different.

miR-name	Control		Overfed		Fold-change
	Expression	Percent	Expression	Percent	
Aan-miR-122-5p	593522.14	70.34%	603499.80	72.01%	1.02
Aan-let-7f	106292.53	11.95%	86157.27	9.91%	0.81
Aan-let-7a	60272.23	6.78%	53729.91	6.18%	0.89
Aan-miR-140-3p	8076.96	0.91%	7267.47	0.84%	0.90
Aan-let-7g	6149.29	0.69%	4683.93	0.54%	0.76
Aan-miR-103/107	5457.45	0.61%	6834.39	0.79%	1.25
Aan-let-7b	4977.79	0.56%	5251.53	0.60%	1.05
Aan-miR-101-3p	4461.01	0.50%	3620.18	0.42%	0.81
Aan-miR-199	4345.30	0.49%	3466.22	0.40%	0.80
Aan-miR-222	3016.07	0.34%	8650.00	0.99%	2.87

**Table 2:** The differentially expressed conserved miRNAs. Differentially expressed miRNAs were identified based on FPKM ≥ 100.00 in either of the two groups, fold change ≥ 1.50 or ≤ 0.67, and *P* < 0.001. Hsa, *Homo sapiens*; Gga, *Gallus gallus*; Tgu, *Taeniopygia guttata*.

miR-name	Mature sequences (5′–3′)	Expressed in control	Expressed in overfed	Fold change	Reference
Aan-miR-1285-3p	CTGGGCAACATAGAGAGACGCG	321.92	0.01	< 0.01	hsa
Aan-miR-146a-5p	TGAGAACTGAATTCCATGGGTTG	305.28	134.97	0.44	gga
Aan-miR-30d-5p	TGTAAACATCCCCGACTGGAAG	2734.73	1348.35	0.49	gga
Aan-miR-29a-3p	TAGCACCATTTGAAATCGGTTA	529.00	279.12	0.53	gga
Aan-miR-30a-5p	TGTAAACATCCTCGACTGGAAG	1892.24	1070.02	0.57	gga
Aan-miR-148a-3p	TCAGTGCACTACAGAACTTTGT	410.89	252.18	0.61	gga
Aan-miR-6682a	TGGAACAACGTAGGTAAGGG	975.09	600.20	0.62	gga
Aan-miR-125b-5p	TCCCTGAGACCCTAACTTGTGA	305.57	195.03	0.64	gga
Aan-miR-100-5p	AACCCGTAGATCCGAACTTGTG	535.15	350.84	0.66	gga
Aan-miR-107b	AGCAGCATTGTACAGGGCTTTT	561.12	917.65	1.64	gga
Aan-miR-4508	AGCGGGGCTGGGCGCGCGC	546.79	917.48	1.68	hsa
Aan-miR-4492	CGGGGCTGGGCGCGCGCCGCG	224.49	394.48	1.76	hsa
Aan-miR-221-3p	AGCTACATTGTCTGCTGGGTTTC	4504.39	8530.40	1.89	gga
Aan-miR-221-5p	ACCTGGCATACAATGTAGATTT	161.99	315.78	1.95	gga
Aan-miR-184-3p	TGGACGGAGAACTGATAAGGGT	651.63	1296.59	1.99	gga
Aan-miR-193a-5p	TGGGTCTTTGCGGGCGAGATGA	65.60	132.05	2.01	gga
Aan-miR-139-3p	TGGAGATGCGGCCCTGTT	49.05	100.25	2.04	gga
Aan-miR-148b-3p	TCAGTGCATCACAGAACTTGGT	609.20	1331.80	2.19	hsa
Aan-miR-375	TTTGTTCGTTCGGCTCGCGTTA	59.06	144.42	2.44	gga
Aan-miR-222	AGCTACATCTGGCTACTGGGTC	6013.67	17262.37	2.87	gga
Aan-miR-203a	GTGAAATGTTTAGGACCACTTG	74.16	403.57	5.44	gga
Aan-miR-2981	GCTGGGCCGGTCGGGCTGGG	0.01	196.09	> 50.00	tgu
Aan-miR-3591-5p	TTTAGTGTGACAATGGTGTTTGA	0.01	134.26	> 50.00	hsa
Aan-miR-3135b	GGCTGGTCCGAGTGCAGTGGTG	0.01	329.73	> 50.00	hsa
Aan-miR-6682b	TGGAGCATCGTAGGTAAGGG	0.01	616.10	> 50.00	gga

### Validation of selected miRNAs by qRT-PCR

**Figure 3:**
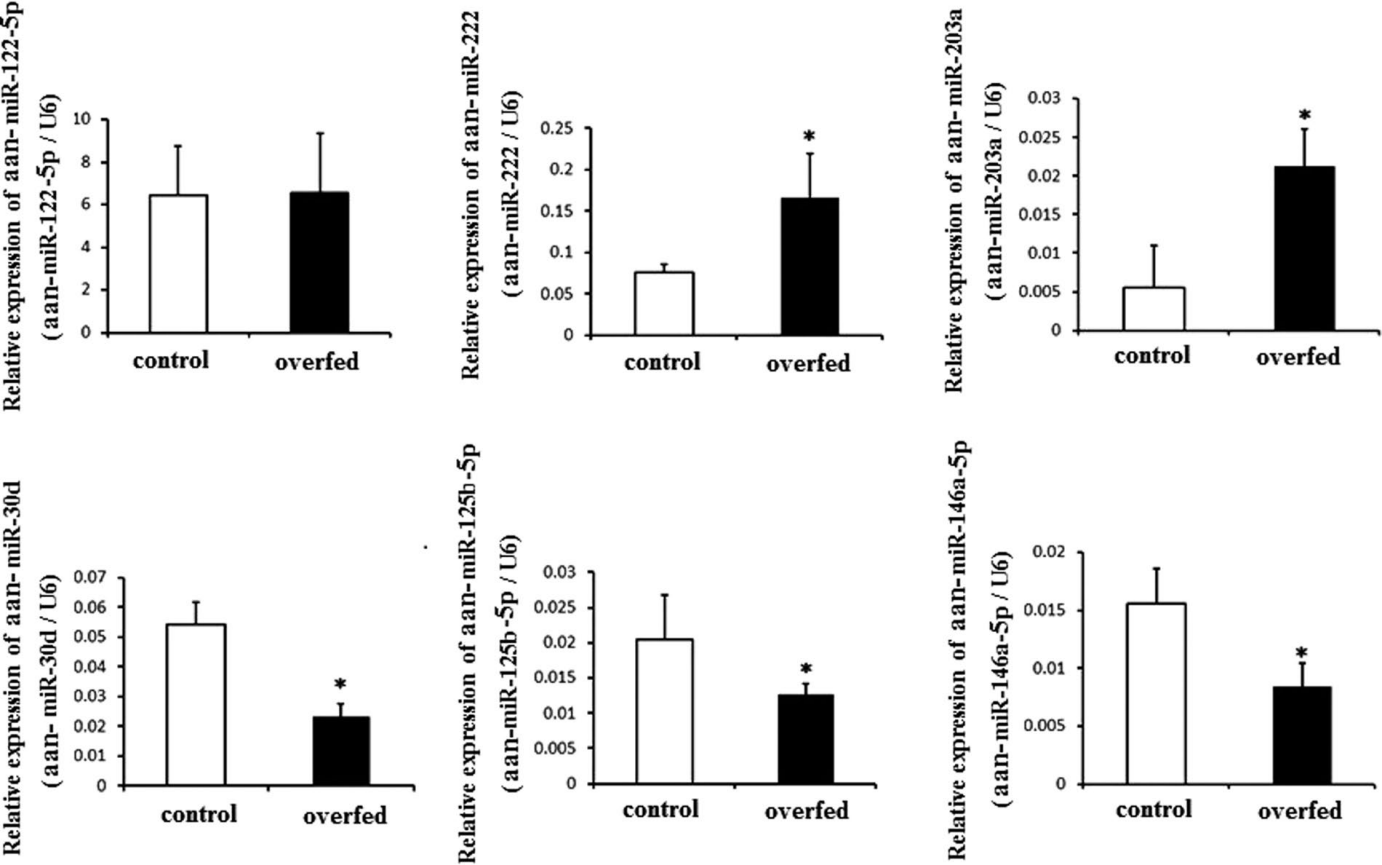
Validation of selected miRNAs by qRT-PCR. *Means *P* < 0.05.

We confirmed 6 miRNAs using qRT-PCR (Fig. 3). Aan-miR-122-5p, the most abundant miRNA in the liver, had similar level in the two groups. Aan-miR-222 was the most abundant upregulated miRNA in overfed geese liver. The relative expression of aan-miR-222 was increased 2.20-fold after overfeeding, while the relative expression of aan-miR-203a was increased 3.84-fold (*P* < 0.05). Conversely, the levels of aan-miR-30d, aan-miR-125b-5p, and aan-miR-146a-5p in overfed groups were only 42.60%, 62.09%, and 54.08% of those in the control groups (*P* < 0.05), respectively. The fold-change was differential between high-throughput sequencing and qRT-PCR. However, the variation trend was consistent between the two detection methods.

### miRNA function analysis

**Figure 4:**
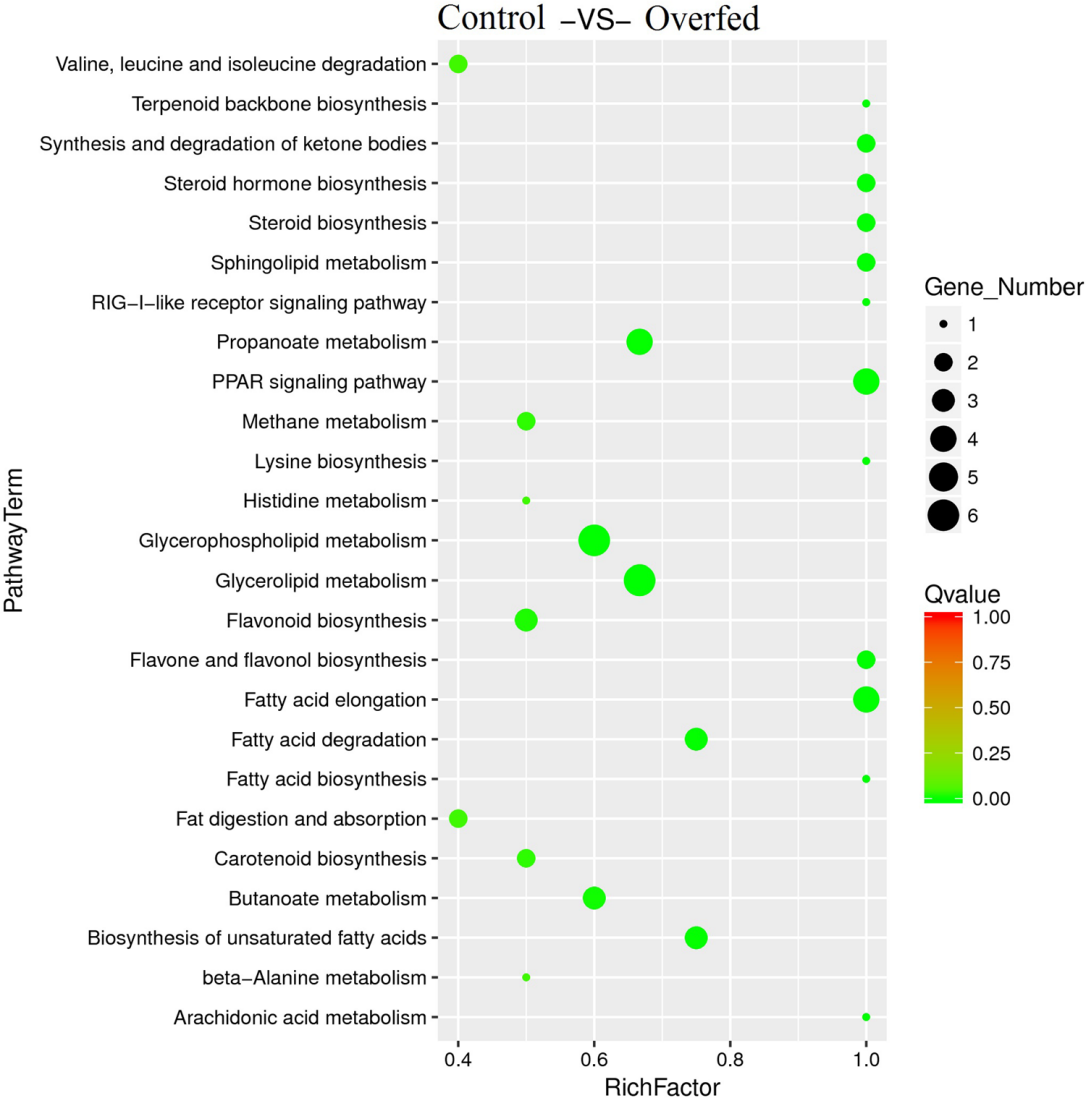
The significantly enriched pathways involved in lipid metabolic. KEGG pathway analysis for the predicted target genes of the differentially expressed miRNAs.

**Figure 5:**
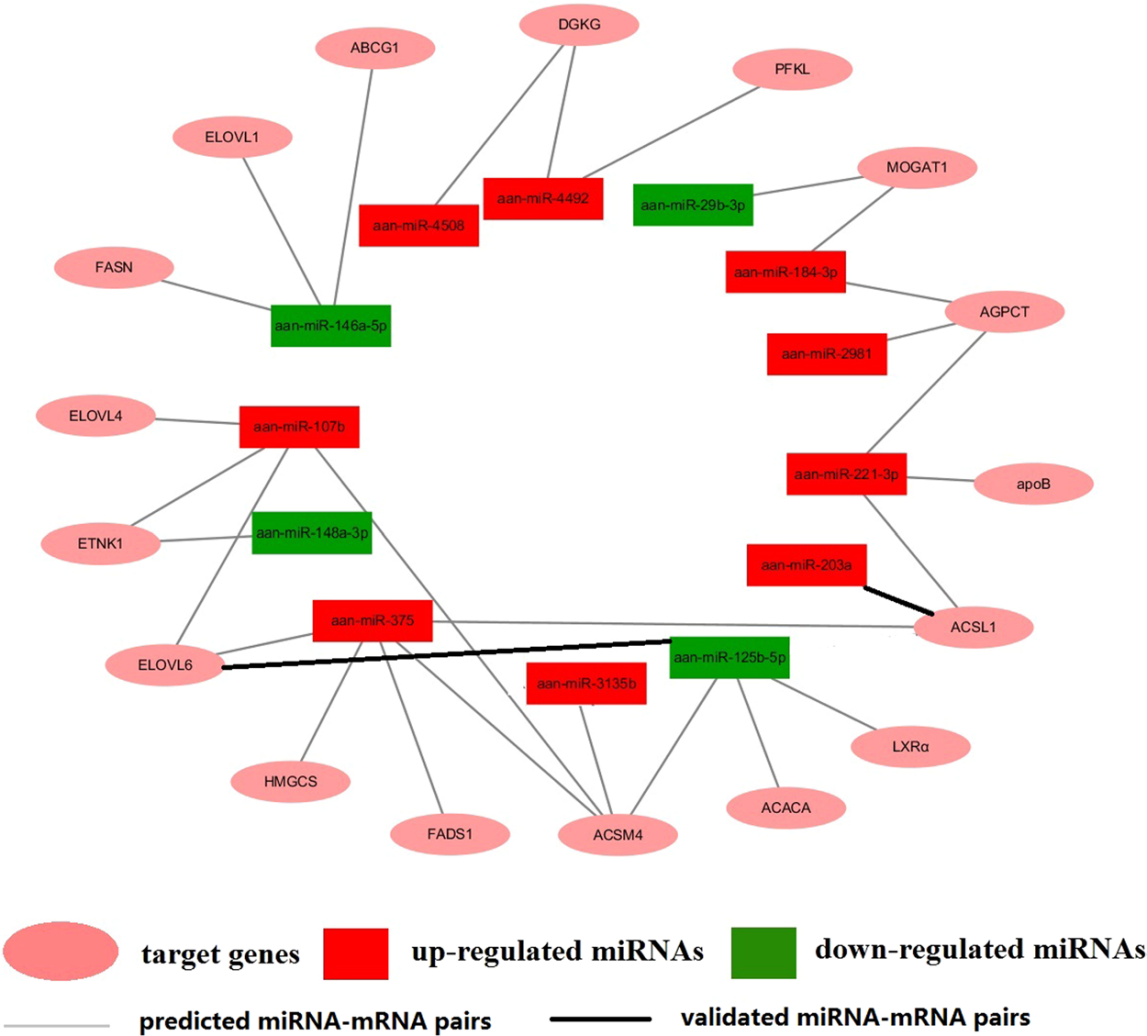
Regulation networks of the differentially expressed miRNAs and their predicted genes involved in lipid metabolism.

**Figure 6:**
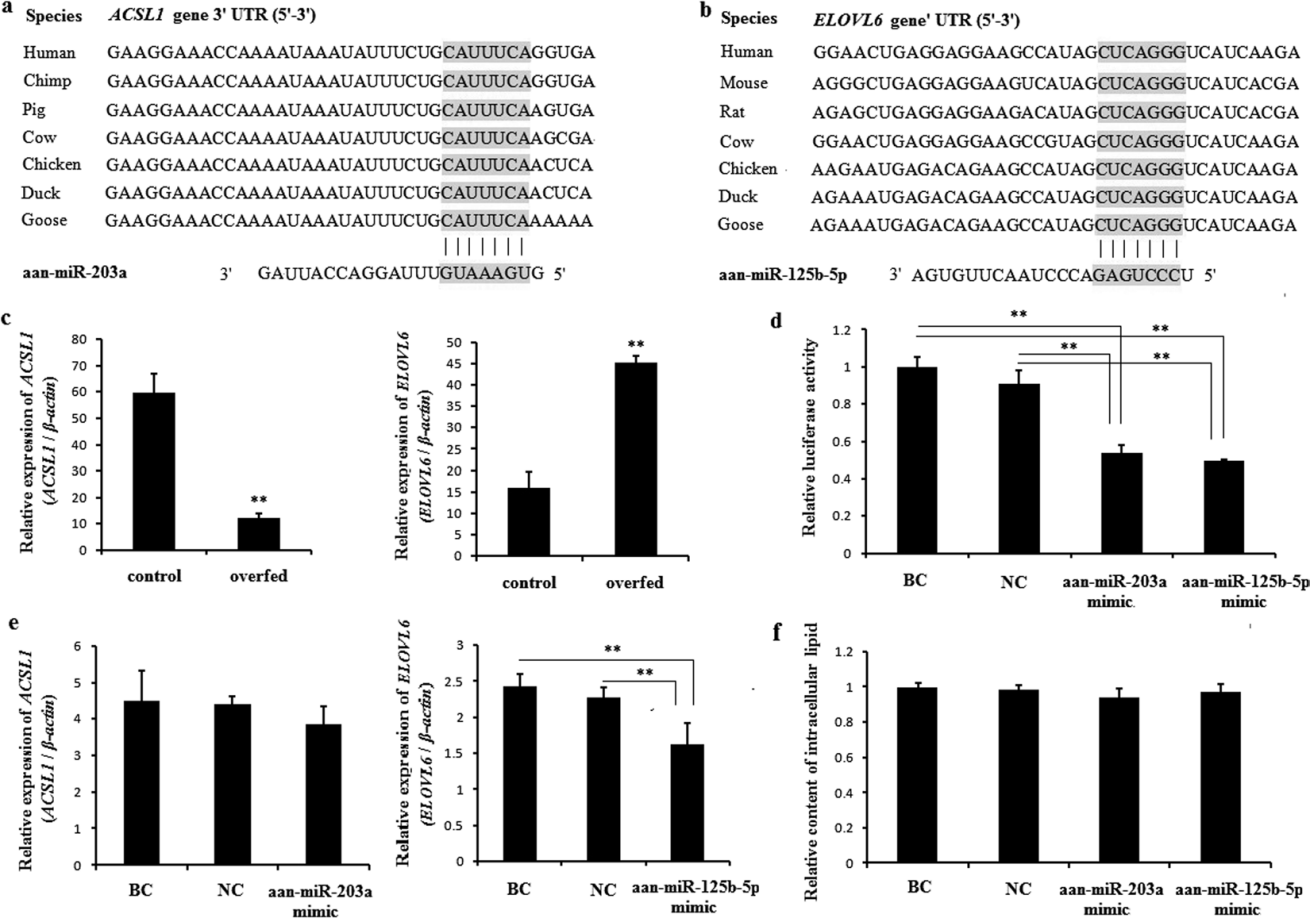
Validation of selected target genes. (**a**) Binding sites for aan-miR-203a in the *ACSL1* 3′UTR of different species; (**b**) Binding sites for aan-miR-125b-5p in the *ELOVL6* 3′UTR of different species; (**c**) Relative expression of *ACSL1* and *ELOVL6* in control and overfed liver samples; (**d**) Luciferase activity assay of the recombined Dual-Luciferase reporter vectors with 3′UTR of *ACSL1* or *ELOVL6* co-transfected with aan-miR-203a mimic or aan-miR-125b-5p mimic; (**e**) Relative expression of *ACSL1* and *ELOVL6* in hepatocytes transfected with aan-miR-203a mimic or aan-miR-125b-5p mimic; (**f**) The relative content of intracellular lipid in hepatocytes transfected with aan-miR-203a mimic or aan-miR-125b-5p mimic. **Means *P* < 0.01.

To understand the biological functions of miRNAs in *A. anser*, we analyzed the putative target genes of differentially expressed miRNAs and classified the putative targets according to their pathways by KEGG pathway analyses. Figure 4 shows some pathways related to lipid metabolism. The network of some miRNAs and their predicted genes involved in glucose and lipid metabolism were shown in Fig. 5. *ACSL1* and *ELOVL6* transcripts may be the target genes of aan-miR-203a (Fig. 6a) and aan-miR-125b-5p (Fig. 6b), respectively. *ACSL1* and *ELOVL6* had opposite expression patterns with aan-miR-203a and aan-miR-125b-5p (Fig. 6c) after overfeeding, respectively. The recombined reporter vectors with the 3′-UTRs of *ACSL1*/*ELOVL6* were co-transfected into goose primary hepatocytes with aan-miR-203a/aan-miR-125b-5p mimic, respectively. The luciferase activities of the two recombined reporters were both significantly suppressed by aan-miR-203a/aan-miR-125b-5p (*P* < 0.05) (Fig. 6d). Compared with blank control (BC) and negative control (NC), the mRNA expression level of *ELOVL6* decreased in goose primary hepatocytes at 48 h after transfection with the aan-miR-125b-5p mimic. And *ACSL1* mRNA level had no significant change in hepatocytes transfected with miR-203a mimic (Fig. 6e). The intracellular lipid contents of hepatocytes transfected with the aan-miR-203a mimic or aan-miR-125b-5p mimic had no significant difference compared with BC group and NC group (Fig. 6f).

## Discussion

To date, many researchers have focused on liver diseases in mammals^11^, such as nonalcoholic fatty liver disease (NAFLD)^8,12^, alcoholic fatty liver (AFL)^13^, etc. miRNAs have been reported to play important roles in the regulation of glucose and lipid metabolism in the liver^14,15^. Domestic Palmipedes are particularly susceptible to hepatic steatosis, and unlike human NAFLD, the fatty liver is nonpathological and reversible^16^. Goose liver has been widely studied by researchers as a good model to investigate the mechanism of lipid metabolism^17^. Since the miRNAs involved in fatty liver production in geese remain to be elucidated, an analysis of the functional miRNAs involved in goose hepatic steatosis was performed on.

In our study, the size, color, weight and histology of the fatty liver showed that the livers of geese produced obvious hepatic steatosis after 21 days overfeeding. It provides a good animal model for fatty liver research. In addition, our lab possesses the *A. anser* genome database for matching analysis^18^ and the miRNAs profile of goose hypothalamus for reference^19^. It provides a more accurate platform for studying the roles of miRNAs in fatty liver production in geese. In this study, we found that 25 conserved miRNAs were differentially expressed in goose liver after overfeeding. In addition, we verified several miRNAs by qRT-PCR. Although some of the fold changes obtained by qRT-PCR were not identical to those obtained by deep sequencing, the trend in the changes was consistent across the two techniques.

Some dysregulated miRNAs in this study were consistent with known changes observed in other studies focused on lipid metabolism. MIR222 was upregulated in NASH and its predicted target genes are known to be involved in carbohydrate metabolism^20^. MIR221 was upregulated in adipose tissue of obesity individuals^21^. Similarly, the expression of aan-miR-222 and aan-miR-221 was increased as indicated by our study. MIR30d was a key regulator of human adipocyte development^22^. MIR148a controlled the expression of key proteins involved in cholesterol-lipoprotein trafficking^23^. Mir29 was downregulated in fatty livers of ob/ob mice^24^. Consistent with these studies, the expressions of aan-miR-30d, aan-miR-148a and aan-miR-29a significantly reduced in fatty liver of goose.

Aan-miR-122-5p is a miRNA with tissue-specific expression and showed predominance in the liver. Consistent with mammals, it accounted for at least 70% in the two groups. Many studies have suggested that MIR122 is important for the regulation of hepatic lipid metabolism^25^. Mir122 knockdown in mice reduced the cholesterol levels in the circulation and liver, decreased fatty acid synthesis, and increased fatty acid oxidation in the liver^26^. Mir122 was downregulated in rats treated with high-fat diet and enriched with or without fructose^27^. However, in our study, the expression of aan-miR-122-5p showed no significant change. And this result is consistent with the change in duck with high fat diet^28^. MIR375 is involved in the regulation of insulin release and glucose homeostasis^29^. Inconsistent with the underexpression of MIR375 in NASH^30^, aan-miR-375 was significantly increased in the fatty liver of goose. It is worth noting that some miRNAs have different change in same processes in different species. For example, MIR125b was abnormal expression in GBM5 cell line treated with ARA and DHA but not in the other, although all the studied cell lines were glioblastoma cells^31^. These differences can be attributed to the presence of different signaling pathways and can be species-specific. Elucidation of the specific functions of these dysregulated miRNAs in the development of goose fatty liver requires further experimental data.

In order to understand the biological function of differential expression miRNAs, we predicted their target genes and classified these genes according to their pathways by KEGG pathway analyses. Many of these target genes play key roles in metabolic pathways leading to the development of hepatic steatosis in the goose fatty liver. Mir203a can be used as the potential biomarker for type 2 diabetes^32^ and alcoholic steatohepatitis^33^ in rat models. Mir125b has been shown related to adipocyte differentiation and adiposity^34,35^. However, few studies described their function in hepatic lipid metabolism. *ACSL1* and *ELOVL6* are predicted target genes of aan-miR-203a and aan-miR-125b-5p, respectively. ACSLs play a critical role in the first committed step in fatty acid metabolism by the thioesterification of long-chain fatty acids into their acyl-CoA derivatives^36^. ELOVL6 is responsible for the elongating C12-C16 to longer fatty acids^37^. In addition, *ACSL1* and *ELOVL6* had opposite expression patterns with aan-miR-203a and aan-miR-125b-5p, respectively. Relative luciferase activity values of recombined Dual-Luciferase reporter vectors with 3′-UTR of *ACSL1* and *ELOVL6* were significantly decreased by aan-miR-203a and aan-miR-125b-5p mimics, respectively. These results suggest that aan-miR-203a and aan-miR-125b-5p may implicate in the regulation of hepatic lipid metabolism by targeting *ACSL1* and *ELOVL6*, respectively. But because anti-ELOVL6 and anti-ACSL1 antibodies specific for goose were lacking, the effect of miRNAs on the protein expression level of their target genes were not measured. However, the overexpression of aan-miR-203a and aan-miR-125b-5p in primary hepatocyte had no significant impact on lipid accumulation. Therefore, the specific function of aan-miR-203a and aan-miR-125b-5p on lipid metabolism of goose need further research.

Goose liver can be a good model to investigate the mechanism of lipid metabolism. In summary, we built fatty liver model of the *A. anser* and identified some conserved miRNAs with significant differences in expression in the fatty liver that may play an important role in hepatic steatosis. These results add to our current understanding of the regulation network in goose lipid metabolism.

## Materials and Methods

### Ethics Statement

All experimental protocols were approved by Animal Ethics Committees of Nanjing Agricultural University and Zhejiang Academy of Agricultural Sciences, China. The slaughter and sampling procedures complied with the “Guidelines on Ethical Treatment of Experimental Animals” (2006) No. 398 set by the Ministry of Science and Technology, China. All efforts were made to minimize animal suffering.

### Animals and experiment design

The experiment was carried out using 90 days-old male Landes Geese hatched on the same day. 60 animals were divided into two groups and bred under natural conditions of light and temperature. All geese were housed in individual cages during the trial period. The carbohydrate-rich diet contains 3500 kcal, 4.8 g fat, and 100 g protein/kg. For 1–2 days of overfeeding, the animals were fed 150 g each time and 2 times per day. For 3–7 days of overfeeding, the animals were fed 150 g at a time and 3 times per day. At the end of overfeeding, they were fed 300 g at a time and 4 times per day. As control, geese were given a growing diet (containing 2800 kcal and 150 g protein/kg) and ad libitum during the trial period. Animals had free access to water at all times. Ten liver samples, including five control and five experimental geese (overfed for 21 d), were prepared for histological studies and analyzed for the expression of miRNAs. Prior to sampling, the geese underwent fasting for 12 h and only water was provided. The workflow can be found as Supplementary Figure S1.

### Histological studies

The geese were overfed for 21 days and the control geese were sacrificed at the same time. The livers of 10 geese were collected and fixed in formalin solution for 24 h, and then embedded in paraffin and sectioned. The slices were stained with hematoxylin-eosin (HE) and observed by microscopy.

### High-throughput sequencing and computational analysis

Total RNA of each sample was extracted with Trizol (Invitrogen, USA). Using a Novex 15% TBE-Urea gel, 20–30-nt long RNA fragments were isolated and purified from 10 μg of total RNA. The small RNA molecules were prepared for high-throughput sequencing according the method described previously^19^. High-throughput sequencing was performed with Illumina HiSeq. 2000 system. After removing the adaptors, low quality tags and contaminants reads, the clean reads were aligned with the goose genome (https://www.ncbi.nlm.nih.gov/nuccore/AOGC00000000). Then we filtered the known non-miRNA reads, such as rRNA, tRNA, snRNA, and snoRNA by screening against ncRNA deposited in the GenBank and Rfam databases. The remaining reads were searched against the miRbase 21 to identify the conserved miRNAs (http://www.miRbase.org/). The conserved miRNAs were named according to the guidelines for the miRNA terminology^38^. The expressions of miRNAs were normalized by Fragments per Kilobase of Transcript per Million Fragments Mapped (FPKM)^39^. Differentially expressed miRNAs were identified based on FPKM ≥ 100.00 in either of the two groups, fold change ≥ 1.50 or ≤ 0.67, and *P* < 0.001. Potential target genes of differential expressed miRNAs were predicted by matching miRNA-3′-UTR sequence and assessing their energy stability using Miranda algorithm^40^. And then GO Term (http://geneontology.org/) and KEGG pathway analyses (http://www.genome.jp/kegg/) of target genes were performed. The miRNA-mRNA interaction network was constructed using Cytoscape 3.5.1 software.

### Validation of miRNAs by qRT-PCR

Total miRNAs from the livers were extracted using the miRcute miRNA isolation kit (TIANGEN, China). miRNAs were reverse transcribed to cDNA with the miRcute miRNA First-Strand cDNA Synthesis Kit (TIANGEN, China) and specific stem-loop primers. mRNAs were reverse transcribed to cDNA with PrimeScript RT reagent Kit with gDNA Eraser (Takara, Japan). Primers used in the qRT-PCR were listed in Supplementary Table S1. qRT-PCR was performed on ABI 7300 system (Applied Biosystems, CA) with THUNDERBIRD SYBR qPCR Mix (TOYOBO, Japan). U6 and β-actin were used as the reference gene, respectively. The relative expression levels of miRNAs and mRNAs were indicated by 2^−△Ct^.

### Dual-luciferase reporter assay

The 3′-UTRs of goose *ACSL1* and *ELOVL6* were amplified and recombined to pmirGLO Dual-Luciferase miRNA Target expression Vector (Promega, USA) according to the manufacturer’s introductions. The verified vector plasmid was co-transfected into goose primary hepatocytes with miRNA mimics or negative control (GenePhama, China) by lipofectamine 2000 (Life Technologies, USA). After 48 h of transfecting, the luciferase activities were measured with Dual-Luciferase Reporter Assay System (Promega, USA).

### Cell culture and transfection

Goose hepatocytes were isolated from 1–2 week-old Landes goose following the perfusion method^41^. Cells were cultured in RPMI1640 medium containing 10% FBS (Gibco, USA) and incubated at 5% CO2, and 37 °C on 24-well cell culture dishes (Sangon, China) at a density of 3 × 105/cm^2^. Medium was changed every 24 h. Cells were distributed into 4 groups after 24 h growth: blank control group (BC), negative control group (NC), aan-miR-203a mimic group and aan-miR-125b-5p mimic group. Transfections were performed using Lipofectamine 2000 (Invitrogen, USA) according to the manufacturer’s introductions. Cells were collected after 48 h of tranfection to detect the expression of *ACSL1* and *ELOVL6*. The intracellular lipid contents of hepatocytes were monitored using multimode reader (Perkinelmer, Singapore) after Nile Red staining.

### Statistical analysis

Differences between two groups were analyzed using the SPSS software based on two-tailed Student’s *t*-test. Differences were considered significant when *P* < 0.05. All data are expressed as the mean ± standard deviation (S.D.).

### Data availability

Our miRNAs dates of high-throughput sequencing are available in NCBI (GEO99200, https://www.ncbi.nlm.nih.gov/geo/query/acc.cgi?acc = GSE99200). All data generated or analyzed during this study are included in this published article (and its Supplementary Information files).

## Electronic supplementary material

supplementary information
