# Supplementary material for: Identification of differentially expressed miRNAs in the fatty liver of Landes goose (Anser anser)
Source: Sci Rep. 2017 Nov 24;7:16296. doi: 10.1038/s41598-017-16632-7 (PMC5701175; doi:10.1038/s41598-017-16632-7)
Supplement: Supplementary file 1 — supplementary information [file 41598_2017_16632_MOESM1_ESM.pdf]

Supplementary Information for:

Identification of differentially expressed miRNAs in the fatty liver of Landes goose (*Anser anser*)

Fang Chen[1, 2+], Hao Zhang[1, 2+], Jinjun Li[2], Yong Tian[2], Jing Xu[2], Li Chen[2], Jintao Wei[1], Na Zhao[1], Xuehai Yang[1], Wei Zhang[1], Lizhi Lu[2*]

[1]Institute of Animal Husbandry and Veterinary Science, Hubei Academy of Agricultural Sciences / Hubei Key Laboratory of Animal Embryo Engineering and Molecular Breeding, Wuhan, 430064, PR China

[2]Institute of Animal Husbandry and Veterinary Science, Zhejiang Academy of Agricultural Sciences, Hangzhou, 310021, PR China

[*]Corresponding author. 1582121877@qq.com; lulizhibox@163.com

[+]These authers contributed equally to this work

Supplementaey Table S1. Distribution of small RNA reads in the sequenced goose liver small RNA library.

category	Control		Overfed	
	total sRNA	percent (%)	total sRNA	percent (%)
exon_antisense	297	0.00%	338	0.00%
exon_sense	51131	0.49%	63172	0.56%
intron_antisense	2485	0.02%	2849	0.03%
intron_sense	18229	0.18%	19433	0.17%
miRNA	9244896	88.92%	9847086	86.98%
rRNA	532164	5.12%	642849	5.68%
repeat	699	0.01%	752	0.01%
scRNA	6536	0.06%	5388	0.05%
snRNA	3540	0.03%	3380	0.03%
snoRNA	5091	0.05%	3129	0.03%
tRNA	109978	1.06%	168996	1.49%
unann	421931	4.06%	563901	4.98%
total	10396977	100%	11321237	100%
genomic	8696250	83.64%	9573773	84.56%

Supplementary Table S2. Detailed information of primers.

miR name	Primer Sequence
miR-122-5p-PF	CTGGTAGGTGGAGTGTGACAATGGT
miR-122-5p-LOOP	CTCAACTGGTGTCGTGGAGTCGGCAATTCAGTTGAGACAAACAC
miR-222a-PF	CTGGTAGGAGCTACATCTGGCTACT
miR-222a-LOOP	CTCAACTGGTGTCGTGGAGTCGGCAATTCAGTTGAGGAGACCCA
miR-30d-PF	CTGGTAGGTGTAAACATCCCCGACT
miR-30d- LOOP	CTCAACTGGTGTCGTGGAGTCGGCAATTCAGTTGAGCTTCCAGT
miR-125b-5p-PF	CTGGTAGGTCCCTGAGACCCTAACT
miR-125b-5p- LOOP	CTCAACTGGTGTCGTGGAGTCGGCAATTCAGTTGAGTCACAAGT
miR-146a-5p-PF	CTGGTAGGTGAGAACTGAATTCCAT
miR-146a-5p- LOOP	CTCAACTGGTGTCGTGGAGTCGGCAATTCAGTTGAGAACCCATG
miR-203a-PF	CTGGTAGGGTGAAATGTTTAGGACC
miR-203a- LOOP	CTCAACTGGTGTCGTGGAGTCGGCAATTCAGTTGAGCAAGTGGT
Universal PR	TCAACTGGTGTCGTGGAGTCGGC
U6 PF	TGGAACGATACAGAGAAGATTAGC
U6 PR	AACGCTTCACGAATTTGCGT
ACSL1-PF	AGCCAACTGTATTTCCTGT
ACSL1-PR	CATCAGCCTTACTCTTCCT
Elovl6-PF	CGTCTTCAGAATACTTGGTG
Elovl6-PR	GTTCGGGTGCTTTGCTTAG
β-actin-PF	GCTTCTCCTTGATGTCACGG
β-actin-PR	CCATCTATGAGGGCTACGCT

Note: PF was upstream primer. PR was downstream primer.

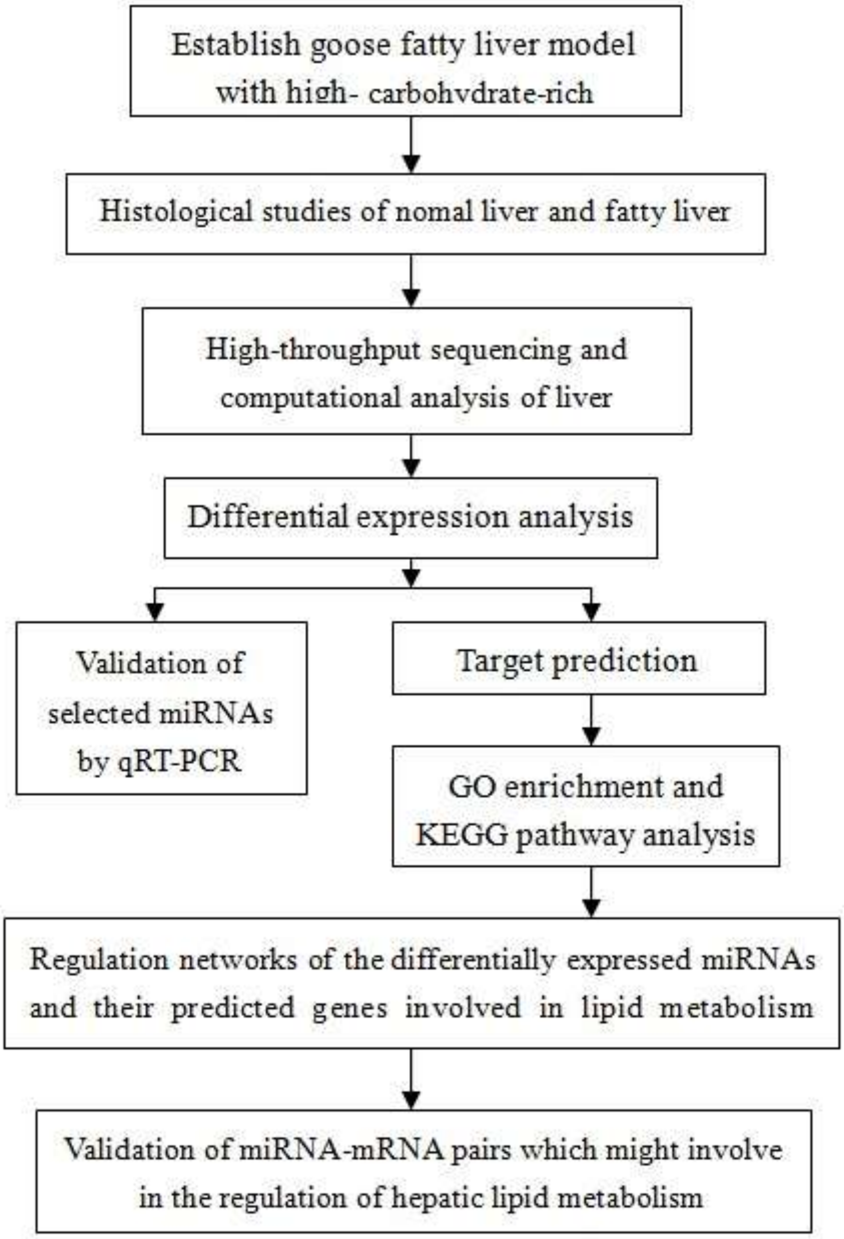

Supplementary Figure S1. The workflow of study

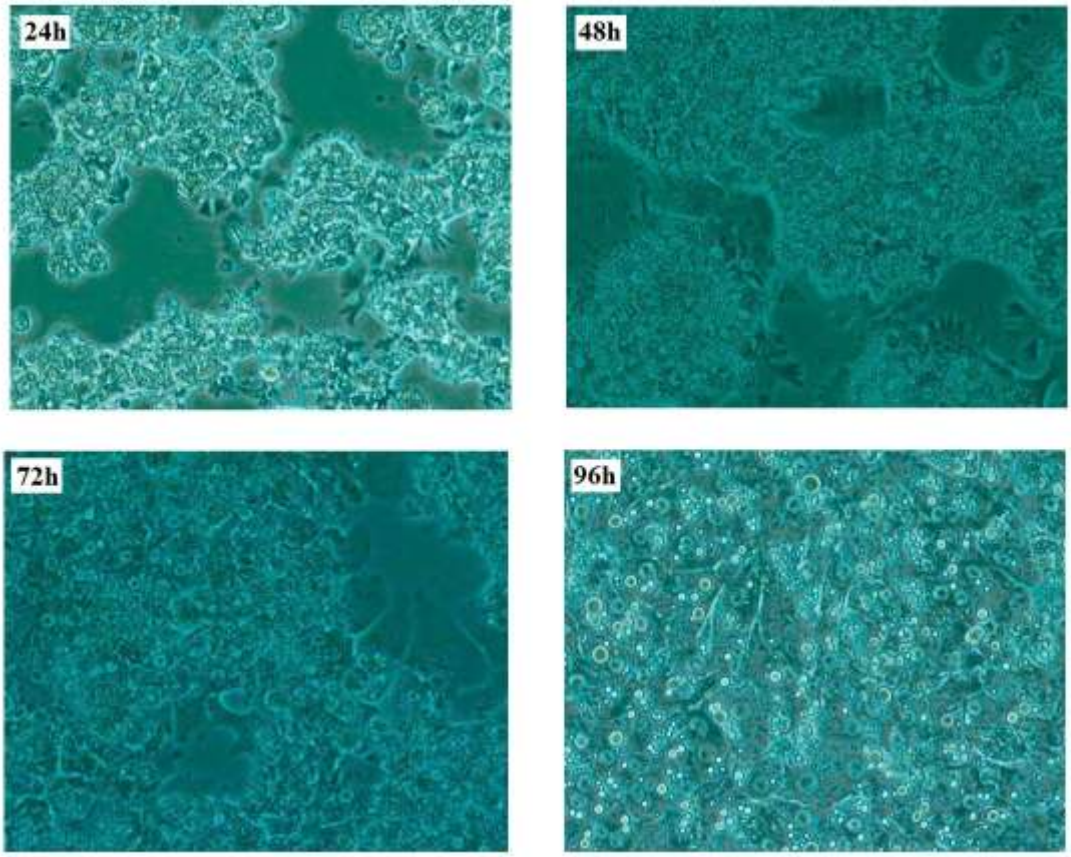

Supplementary Figure S2. Primary hepatocyte of goose (100×). Hepatocytes were isolated for Landes (age of 1-2 week) following the method of Seglen (1976)
